# Machine learning-based prediction of SARS-CoV-2 bioactivity: integrating IC_50_ regression and activity classification using multi-task neural networks

**DOI:** 10.1186/s12859-026-06573-2

**Published:** 2026-08-05

**Authors:** Aya I. Maiyza, Sohila Osama, Hanan A. Hassan

**Affiliations:** 1 Informatics Research Institute (IRI), City of Scientific Research and Technological Applications (SRTA-City), Alexandria, Egypt; 2 Institute of Graduate Studies and Research, Alexandria University, Alexandria, Egypt

**Keywords:** SARS-CoV-2, Bioactivity prediction, IC_50_ modeling, Machine learning, Multi-task neural networks, QSAR, Ligand efficiency, ChEMBL

## Abstract

Accurate prediction of compound bioactivity is essential for accelerating antiviral drug discovery and reducing experimental costs. Machine learning (ML) methods have shown considerable promise in modeling structure–activity relationships and compound potency. In this study, we present an integrated ML framework for predicting IC_50_ and pIC_50_ values of compounds active against SARS-CoV-2, key indicators of antiviral potency. The proposed framework comprises three complementary approaches: (i) a regression model for quantitative IC_50_ prediction validated against experimental data; (ii) a classification model that categorizes compounds into active and inactive classes to support compound prioritization; and (iii) a multi-task neural network that jointly performs IC_50_ regression and activity classification, enhancing predictive performance and interpretability. A distinctive feature of this work is the incorporation of ligand efficiency (LE) as a criterion for activity classification, offering an alternative perspective on compound prioritization that has not been previously explored in SARS-CoV-2 bioactivity modeling. The proposed models demonstrate strong predictive capability, achieving a coefficient of determination ($R^2$) of 0.77 using a neural network with feature selection, while the Random Forest classifier attains an accuracy, precision, and recall of approximately 0.92. These results highlight the potential of integrated regression, classification, and multi-task learning approaches as scalable and cost-effective tools for SARS-CoV-2 bioactivity prediction and antiviral drug discovery.

## Introduction

Drug discovery is a complex and resource-intensive process that involves the identification and development of compounds with therapeutic potential against diverse diseases. This multifaceted pipeline typically includes target identification, compound screening, and preclinical evaluation, where the identification of potent and selective compounds plays a critical role in determining downstream success. Systematic screening and optimization strategies aim to identify molecules with favorable pharmacological profiles while minimizing adverse effects [1].

The emergence of the SARS-CoV-2 pandemic has prompted an unprecedented global effort to accelerate the discovery and development of effective antiviral therapeutics [2, 3]. In response, pharmaceutical companies and research institutions have increasingly adopted computational and data-driven approaches to complement experimental screening and reduce development timelines.

The half-maximal inhibitory concentration (pIC$_{50}$) is a fundamental metric in drug discovery, representing the negative logarithm of the concentration required to inhibit 50% of a biological response. pIC$_{50}$ serves as a key indicator of compound potency and provides quantitative insight into structure–activity relationships. In the context of SARS-CoV-2, accurate prediction of pIC$_{50}$ values is particularly important for prioritizing compounds with potential antiviral activity and guiding lead optimization efforts [2]. Traditional computational approaches, such as quantitative structure–activity relationship (QSAR) modeling, have long been employed to predict pIC$_{50}$ by correlating molecular descriptors with biological activity using statistical and machine learning techniques [2, 4].

QSAR and machine learning models have been successfully applied across a wide range of therapeutic areas, including antibacterial [5], anticancer [6, 7], and antiviral drug discovery [8, 9]. Recent advances in machine learning have further enhanced predictive performance, enabling both IC$_{50}$ regression [8, 10] and activity classification for identifying promising chemotypes against viral targets [11].

Despite these advances, experimental determination of IC$_{50}$ values remains costly, time-consuming, and associated with biosafety constraints, particularly for emerging viral pathogens. These challenges underscore the need for reliable, scalable, and cost-effective computational frameworks that can support compound prioritization and reduce experimental burden.

Building upon our previously published work [12], which demonstrated the application of analytical and machine learning techniques in SARS-CoV-2 drug discovery, the present study extends this effort by developing integrated and scalable predictive models for compound potency and activity classification. The key contributions of this work are summarized as follows:

- *Regression approach* We develop a machine learning regression model for predicting IC$_{50}$ values of compounds active against SARS-CoV-2. Model performance is validated using published experimental data, demonstrating its potential to reduce the cost, time, and experimental risks associated with IC$_{50}$ determination. A web-based application is also provided to facilitate rapid pIC$_{50}$/IC$_{50}$ prediction.
- *Classification approach* We propose a binary classification framework that categorizes compounds into active and inactive classes, supporting efficient prioritization for experimental validation and antiviral drug development.
- *Multi-task neural network (MTNN) approach* We introduce a multi-task neural network that jointly performs IC$_{50}$ regression and activity classification. By integrating both tasks within a unified learning framework, the proposed MTNN improves predictive performance and provides more comprehensive insights into compound bioactivity.

The remainder of this paper is organized as follows: Section “Related work” reviews related studies on QSAR modeling and machine learning approaches for pIC$_{50}$/IC$_{50}$ prediction. Section “Methodology” describes the dataset, feature engineering, and modeling methodology. Section “Results and discussions” presents the experimental results and model evaluation. Finally, Section “Conclusion and future work” concludes the paper and outlines future research directions.

## Related work

Computational modeling has played an increasingly important role in modern drug discovery, particularly in the prediction of compound activity and potency. Among these approaches, quantitative structure–activity relationship (QSAR) modeling has been widely used to estimate the biological activity of compounds across a variety of therapeutic areas, including antibacterial, anticancer, and antiviral drug discovery. QSAR models typically rely on molecular descriptors to establish quantitative relationships between chemical structure and biological response [5–7].

In recent years, machine learning (ML) techniques have emerged as powerful extensions of traditional QSAR modeling, enabling the capture of complex and non-linear relationships between molecular features and biological activity. Several studies have employed ML algorithms such as support vector machines (SVM), random forests (RF), and neural networks to predict IC$_{50}$ values, a key indicator of compound potency [8, 10]. These approaches have generally demonstrated improved predictive accuracy and generalization compared to classical QSAR methods. In addition to regression-based modeling, ML techniques have also been applied to classify compounds as potential inhibitors of viral proteases, thereby facilitating the identification of promising antiviral chemotypes [11].

The COVID-19 pandemic further emphasized the need for reliable and rapid computational approaches to identify potential therapeutic candidates. Numerous studies have leveraged QSAR and ML-based models to prioritize compounds for SARS-CoV-2 targets, including the viral main protease and spike protein [2, 3]. These efforts aimed to accelerate virtual screening pipelines, reduce experimental costs, and mitigate biosafety risks associated with large-scale experimental testing.

Recent methodological advancements have focused on ensemble learning strategies, which combine predictions from multiple base models to enhance robustness and predictive performance. Ensemble techniques, such as bagging and boosting, have proven effective in reducing model variance and improving generalization, particularly when applied to chemically diverse datasets [4].

Despite these advances, several challenges remain. Many existing studies focus on either IC$_{50}$ regression or activity classification as independent tasks, limiting the ability to exploit shared information between quantitative potency prediction and categorical activity assessment. In addition, issues related to data quality, model interpretability, and the integration of domain-specific knowledge continue to constrain the broader applicability of ML-based QSAR models in drug discovery.

In the context of SARS-CoV-2, accurate prediction of pIC$_{50}$/IC$_{50}$ values remains essential for the rapid identification and prioritization of potent antiviral compounds. While experimental assays are indispensable, their high cost and time requirements highlight the need for scalable computational alternatives. Table 1 summarizes representative studies in computational drug discovery, categorizing commonly used modeling techniques, application domains, and key contributions. In contrast to prior studies that primarily address IC$_{50}$ regression or activity classification as separate tasks, the present work introduces an integrated multi-task learning framework that jointly models quantitative potency and categorical activity, enabling a more comprehensive assessment of SARS-CoV-2 bioactivity.

**Table 1:** Taxonomy of related work in computational drug discovery

Authors	Category	Technique	Focus Area	Key Contributions
Pir et al. [5]	Traditional QSAR	Statistical regression	Antibacterial	Models for pyrazole derivatives
Suvannang et al. [6]	Traditional QSAR	Descriptor-based models	Anticancer	Discovery of high-potency compounds
Worachartcheewan et al. [9]	Traditional QSAR	Molecular descriptors	Antiviral	Large-scale dataset modeling
Ekosputri et al. [4]	Machine learning-based QSAR	Random forests (RF)	IC$_{50}$ prediction	Accurate SARS-CoV-2 compound predictions
Mekni et al. [11]	Machine learning-based classification	Support vector machines (SVM)	SARS-CoV-2 protease inhibitors	Classification of active chemotypes
Ferdous et al. [8]	Deep learning-based QSAR	Deep learning frameworks	SARS-CoV-2 main proteases	IC$_{50}$ prediction for SARS-CoV-2 main proteases
Aitouhanni and Berqia [2]	Comparative AI analysis	ML versus DL methods	COVID-19	Evaluation of ML and DL methods
Jiménez-Luna et al. [3]	AI-driven virtual screening	AI-based virtual screening	COVID-19	Identification of potential inhibitors
Nand et al. [10]	Feature engineering for QSAR	Feature engineering	IC$_{50}$ prediction	Enhanced virtual screening
Our approach	Machine learning-based QSAR regression and classification	Deep neural networks with feature engineering	IC$_{50}$ prediction and activity classification	Integrated multi-task framework for SARS-CoV-2 bioactivity prediction

## Methodology

This section presents the methodology used in the study, detailing the processes and techniques used to develop and evaluate the bioactivity prediction models. Subsection “Dataset” provides an overview of the dataset used in the experiments, while Subsection “Data preprocessing and bioactivity classification” explains the data preprocessing and bioactivity classification procedures. Section “Activity prediction approaches” discusses the different activity prediction approaches utilized, including both regression and classification models. In Subsection “Performance metrics”, the performance metrics used to evaluate the model outcomes are presented. Finally, Subsection “Bioactivity prediction web application” introduces the bioactivity prediction web application, designed to provide an interactive platform for users to leverage the developed models in drug discovery efforts.

### Dataset

The bioactivity data used in this study were sourced exclusively from the ChEMBL database, a widely recognized resource containing experimental results for a vast array of small molecules and their interactions with biological targets [13]. The study focuses on SARS-CoV-2 bioactivity prediction, an important research area given the continuing global impact of coronavirus-related diseases [14].

The dataset was restricted exclusively to SARS-CoV-2 targets obtained from ChEMBL. The included compounds were primarily associated with key SARS-CoV-2 proteins, including replicase polyprotein 1ab and the 3C-like proteinase (Mpro), which play essential roles in viral replication and proteolytic processing. A summary of the SARS-CoV-2 protein targets represented in the dataset and their corresponding compound counts is provided in Table 2.

**Table 2:** Summary of SARS-CoV-2 protein targets included in the dataset

Viral species	Protein target	Number of compounds
SARS-CoV-2	Replicase polyprotein 1ab	118
SARS-CoV-2	3C-like proteinase (Mpro)	1206

Compound counts correspond to the filtered dataset after preprocessing and removal of incomplete records

Data collection from ChEMBL was performed using the following filtering criteria: *organism* = “SARS coronavirus 2”, *assay_type* IN (“B”, “F”), and *target_type* IN (“SINGLE PROTEIN”, “PROTEIN COMPLEX”). Assay types “B” (binding assays) and “F” (functional assays) were retained because both correspond to experimentally validated bioactivity measurements that are widely used in ChEMBL-based drug discovery and QSAR studies [15, 16]. To ensure biological consistency and avoid cross-virus variability in compound activity, the dataset was restricted exclusively to experimentally validated SARS-CoV-2 targets obtained from ChEMBL.

The dataset was further refined to include compounds with experimentally determined IC$_{50}$ values. IC$_{50}$, or the half-maximal inhibitory concentration, is a standard metric in drug discovery that quantifies compound potency by measuring the concentration required to inhibit a biological target by 50%. This metric is widely used in pharmaceutical research as it enables direct comparison of compound efficacy across different molecules [17]. IC$_{50}$ values reported in ChEMBL were retained in micromolar units (*μ*M) to maintain consistency with commonly reported experimental measurements. All pIC$_{50}$ values were calculated directly from IC$_{50}$ (*μ*M) using the transformation $pIC_{50} = -\log _{10}(IC_{50})$. In QSAR modeling, pIC$_{50}$ is commonly used as the target variable due to its favorable numerical properties and interpretability [18].

After applying these filtering criteria, the final dataset comprised 1,803 bioactivity data points, representing a focused collection of compounds relevant to human-related coronaviruses. Notably, the dataset emphasizes two key viral protein targets: replicase polyprotein 1ab and SARS coronavirus 3C-like proteinase. These proteins play essential roles in viral replication and survival [19], making them highly relevant targets for antiviral drug discovery.

By focusing exclusively on IC$_{50}$ values, the dataset ensures consistency across measurements and facilitates a more targeted analysis of compound bioactivity. This streamlined approach reduces variability that could arise from the inclusion of alternative activity metrics, such as $K_i$ values or inhibition percentages, ensuring that the results remain directly comparable and aligned with the objectives of this study. Overall, this refined dataset provides a robust foundation for predictive modeling and computational drug discovery aimed at combating coronavirus-related diseases [20].

### Data preprocessing and bioactivity classification

Several preprocessing steps are taken to refine and normalize the data, ensuring the dataset is appropriate for predictive modeling and bioactivity classification. These steps involve filtering the data based on experimental criteria, addressing missing values, and classifying compounds according to their ligand efficiency.

#### Selection of relevant compounds

The dataset is sourced from the ChEMBL database, specifically targeting compounds associated with human coronavirus proteins. The selection is further refined to include only those compounds with experimentally determined IC50 values, which measure the half-maximal inhibitory concentration of a compound [21]. IC50 is a critical parameter for assessing the potency of small molecules and their ability to inhibit target proteins [22]. This focus on IC$_{50}$ ensures consistency in bioactivity measurements and allows for direct comparisons between compounds.

#### Data cleaning and standardization

The initial dataset is thoroughly cleaned to address missing or inconsistent values. Approximately 3% of the dataset, corresponding to entries with missing IC$_{50}$ values, is removed. Additionally, entries where the standard unit is not nanomolar (nM) are excluded, constituting roughly 1% of the dataset. This standardization ensures that all data points are directly comparable, enhancing the reliability of subsequent analyses.

To prevent computational errors and ensure compatibility with logarithmic transformations, IC$_{50}$ values are normalized. Extremely high values are capped at $10^8$ nM, while entries with zero values are replaced with a minimal threshold of $10^{-12}$ nM. These adjustments ensure that all values remain within a biologically meaningful range.

#### Ligand efficiency calculation

Ligand efficiency (LE) is calculated to assess the effectiveness of compounds relative to their molecular size, represented by heavy atom counts (HAC). LE provides a normalized measure of bioactivity and serves as a critical metric in early drug discovery [23]. The ligand efficiency cutoff is set between 0.2 and 0.35 to identify “hit” compounds with potential for optimization [24]. This range is chosen to account for the diversity in molecular size across the screened compounds, ensuring a broad and chemically diverse set of hits.

The calculated ligand efficiency values facilitate the identification of promising compounds that can serve as starting points for further optimization [25]. Compounds meeting the LE criteria are classified as “active”, while those outside this range are deemed “inactive”. This classification reflects the potential of the compound to bind effectively to the target protein and be optimized into drug-like molecules [26].

#### Bioactivity classification

The classification of compounds into active and inactive categories is based on ligand efficiency (LE) values [27]. In this study, ligand efficiency–based classification is considered the primary strategy for defining bioactivity classes, while IC$_{50}$ Compounds with LE values within the defined range (0.2–0.35) are considered active, indicating measurable activity against the target protein, whereas compounds outside this range are classified as inactive.

The dataset exhibited a moderate class imbalance between active and inactive compounds generated using the 10 ${\upmu }$M threshold, comprising 781 active compounds and 543 inactive compounds. To establish a clearer binary classification framework, intermediate compounds were excluded from the classification task. The final class distribution was evaluated prior to model training to assess imbalance effects [28, 29].

Although ligand efficiency (LE) provides a normalized measure of bioactivity relative to molecular size, it does not fully replace IC$_{50}$-based potency assessment. IC$_{50}$ reflects the absolute inhibitory potency of a compound, whereas LE evaluates size-normalized efficiency and is primarily used during hit prioritization and lead optimization [23]. Therefore, this study integrates both IC$_{50}$-based and LE-based perspectives to provide a more comprehensive evaluation of compound bioactivity and prioritization.

Notably, this classification strategy has not been previously applied in COVID-19 bioactivity research, representing a novel aspect of this study. Compounds classified as active according to the LE criterion are considered potential candidates for hit-to-lead optimization, where chemical modifications can enhance potency and improve drug-like properties. The broader LE range adopted in this work allows for a chemically diverse pool of hit compounds, accommodating variations in molecular size and structural complexity [30].

The final dataset comprises compounds with normalized IC$_{50}$ values, standardized units, and calculated ligand efficiencies. Each compound is assigned a bioactivity class based on the defined criteria, providing a robust foundation for subsequent machine learning analyses and antiviral drug discovery efforts.

#### Lipinski descriptors calculation

Christopher Lipinski, a scientist at Pfizer, proposed a set of rules for evaluating the druglikeness of compounds [31]. The following pharmacokinetic properties determine druglikeness, collectively referred to as ADME: (i) Absorption, (ii) Distribution, (iii) Metabolism, and (iv) Excretion, as outlined by [32].

This encompasses the pharmacokinetic profile of a drug. Lipinski’s analysis of all orally active FDA-approved drugs leads to the formulation of the well-known Rule of Five, or Lipinski’s Rule [33]. These physicochemical properties are critical in determining a drug’s absorption, distribution to target tissues, metabolism, and eventual bodily excretion.

Lipinski’s Rule of Five outlines the key criteria for a compound to be considered a potential drug candidate [34]. These criteria are as follows:

1. Molecular weight should be less than 500 Dalton.
2. The octanol-water partition coefficient (LogP) should be less than 5.
3. The number of hydrogen bond donors should be fewer than 5.
4. The number of hydrogen bond acceptors should be fewer than 10.

#### Morgan fingerprints

Morgan fingerprints are molecular representations commonly used in cheminformatics. They are generated using the Morgan algorithm, which is an iterative procedure that assigns unique identifiers to atoms based on their atomic environment [35]. These fingerprints effectively capture molecular substructures and are widely applied in tasks such as similarity searching, molecular clustering, and ML models for predicting molecular properties [36]. Morgan fingerprints were generated using RDKit with a radius of 2 and a 1024-bit vector representation.

**Fig. 1:**
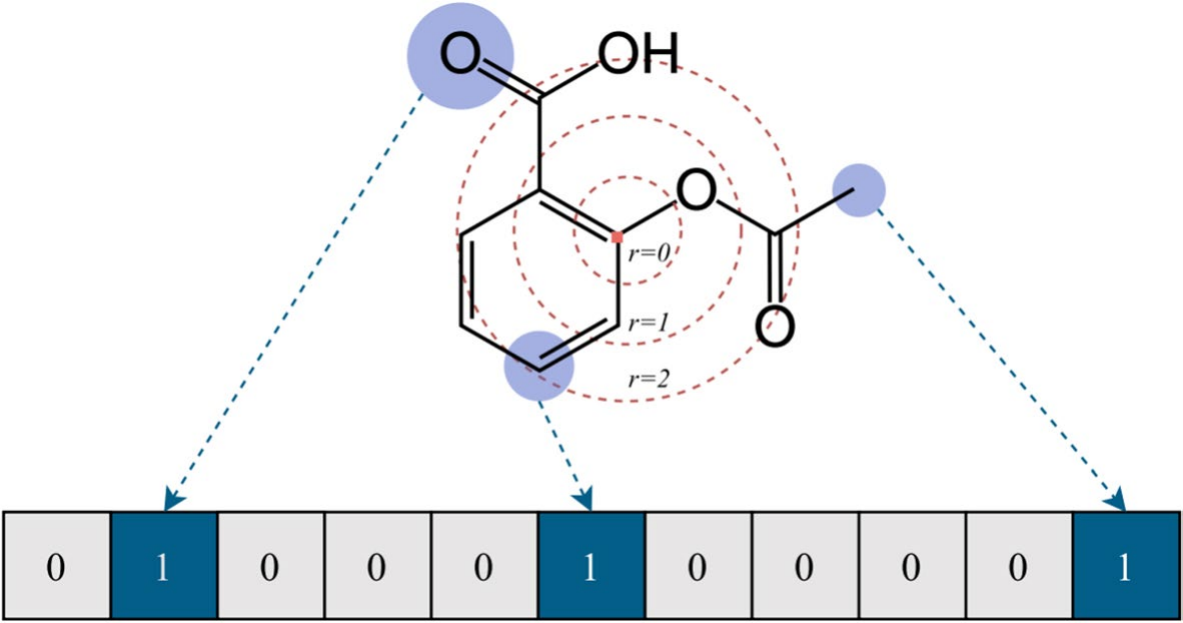
An example of structure-based representations. Morgan fingerprints are a binary vector in which ‘one (1)’ and ‘zero (0)’ indicate the ‘presence’ and ‘absence’ of a defined substructure, respectively. A set of Morgan substructures is determined by the number of selected bits (1024) and radius (r). The size of the substructure is associated with the radius [38]

The Morgan algorithm generates fingerprints by iteratively expanding the atomic environment around each atom up to a specified radius [35, 37]. During each iteration, the algorithm takes into account atomic connectivity and types, encoding this information into a hashed bit vector [35]. This bit vector indicates the presence or absence of specific molecular substructures. A major advantage of Morgan fingerprints is their ability to create highly informative and compact molecular representations, making them ideal for large-scale computational analyses. This process is illustrated in Fig. 1.

In this study, Morgan fingerprints are used to encode molecular features, providing a robust and efficient representation for training our ML models. Their use enables the effective capture of key substructural information, which contributes significantly to the performance of the models in predicting target properties.

### Activity prediction approaches

Various ML techniques are used to develop predictive models that estimate a compound’s potency based on its molecular features. In this study, we adopt three different approaches: (i) regression-based models, (ii) classification-based models, and (iii) multi-task neural network models.

#### Regression approach

In this study, various ML techniques are used to predict the target variable pIC$_{50}$ for regression tasks. The methodology follows a structured approach, beginning with the selection of appropriate libraries and tools. We use pandas, numpy, scikit-learn, xgboost, joblib, shap, and matplotlib for data manipulation, model training, evaluation, and visualization. These tools facilitate efficient data handling, implementation of ML models, and the generation of insights through interpretability techniques.

For data preparation, the feature matrix is constructed by concatenating molecular descriptors from the dataset with Morgan fingerprints. An extra feature, MolWt_LogP, is introduced by multiplying molecular weight and LogP values, aiming to capture potential interactions between these molecular properties. The final dataset defines the target variable as the pIC$_{50}$ value.

We apply feature scaling and selection to ensure consistency and improve model performance. All molecular descriptors and Morgan fingerprint features were normalized using StandardScaler prior to model training to ensure numerical consistency across feature columns. Feature selection is conducted using LassoCV with cross-validation, identifying the most relevant features while reducing dimensionality and preventing overfitting. The selected features are subsequently stored for further analysis and model development.

For feature engineering, we curate a comprehensive set of molecular descriptors, including molecular weight (MolWt), LogP, number of hydrogen bond donors (NumHDonors), number of hydrogen bond acceptors (NumHAcceptors), and Morgan fingerprints. The newly engineered feature, MolWt_LogP, enhances the predictive model by capturing interactions between molecular weight and LogP.

The model training phase involves splitting the dataset into training and testing sets. We implement three regression models: (i) RandomForestRegressor, (ii) GradientBoostingRegressor, and (iii) XGBRegressor. To improve prediction accuracy and robustness, ensemble predictions are generated by averaging the outputs of these models. The trained models are also serialized using joblib, allowing for future reuse and deployment.

#### Classification approaches

For the classification approach, we implement various ML classifiers to predict the bioactivity class of compounds based on IC$_{50}$ and LE criteria. The methodology follows a structured approach, beginning with the selection of appropriate libraries and tools. We utilize pandas, rdkit, scikit-learn, xgboost, numpy, matplotlib, and seaborn for data processing, model training, evaluation, and visualization. These tools facilitate efficient data manipulation, feature extraction, model implementation, and interpretation of results.

The dataset was loaded and preprocessed during the data preparation stage by converting SMILES strings into RDKit molecule objects. Molecular descriptors and Morgan fingerprints were computed to numerically represent the chemical structures. These features were then combined with the original dataset to create a comprehensive input matrix suitable for ML models.

The bioactivity classification is based on the following criteria:

- IC$_{50}$-based classification:
  - *Active* IC50 < 10 $${\upmu }$$M
  - *Inactive* IC50 *\ge* 10 $${\upmu }$$M
- LE-based classification:
  - *Active* 0.2 *łe* LE *łe* 0.35
  - *Inactive* LE < 0.2 or LE > 0.35

The IC$_{50}$ threshold is commonly applied in drug discovery pipelines to filter out weakly active compounds [39], while the LE criteria are used to identify promising candidates for further optimization [23, 24]. ML models leverage these classification criteria to predict the bioactivity of new compounds based on their chemical features.

The dataset includes molecular descriptors such as Molecular Weight (MolWt), LogP, Number of Hydrogen Bond Donors (NumHDonors), and Number of Hydrogen Bond Acceptors (NumHAcceptors), along with fingerprints derived from SMILES data. These features are standardized using standard scaling. Feature selection is conducted using LassoCV, resulting in a refined subset of features for model training and evaluation.

The feature matrix is constructed for feature and target definition by excluding non-informative columns such as the bioactivity class and molecule identifiers. The target variable is defined by encoding bioactivity classes based on IC$_{50}$ and LE thresholds, ensuring suitability for classification tasks.

During the model training phase, the dataset was divided into training and testing sets using an 80:20 split to evaluate model performance on unseen data.

To address class imbalance, SMOTE (Synthetic Minority Oversampling Technique) was applied exclusively to the training set prior to model development, thereby avoiding data leakage [28]. Model development incorporated StratifiedKFold cross-validation (n_splits=5, shuffle=True, random_state=42) on the training data to improve robustness and reduce the risk of overfitting. Following model development, the classifiers were trained using the complete training set and subsequently evaluated on the independent test set using accuracy, precision, recall, F1-score, and AUC metrics.

Various classification algorithms are implemented, including Logistic Regression, Support Vector Classifier (SVC), Decision Tree Classifier, Random Forest Classifier, Gradient Boosting Classifier, AdaBoost Classifier, K-Nearest Neighbors (KNN), Gaussian Naïve Bayes (GaussianNB), and Multi-Layer Perceptron (MLP) Classifier. Each model is trained using the extracted molecular features to predict the bioactivity class of compounds based on the defined IC$_{50}$ and LE criteria.

#### MTNNs approach

**Fig. 2:**
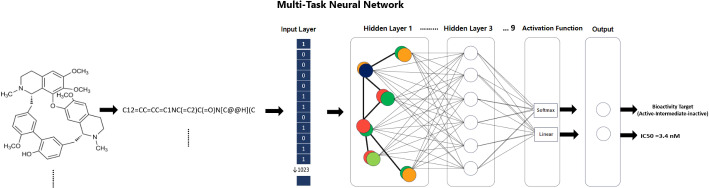
The architecture of the multi-task neural network model

We develop a MTNN model, as shown in Fig. 2, to perform regression and classification tasks simultaneously, enabling efficient learning from molecular features. This approach leverages a shared architecture to extract common representations while optimizing separate objectives for regression and classification.

For libraries and tools, we use TensorFlow, Keras, pandas, scikit-learn, joblib, numpy, and matplotlib, facilitating model development, training, evaluation, and visualization. These libraries provide essential functions for handling data, building deep learning models, optimizing training processes, and analyzing results.

In the data preparation phase, the feature matrix is created by combining molecular descriptors and Morgan fingerprints to represent the structural and physicochemical properties of the compounds. Since neural networks are sensitive to feature scaling, all molecular descriptors and Morgan fingerprint features were normalized using StandardScaler, ensuring stable and efficient model training.

**Fig. 3:**
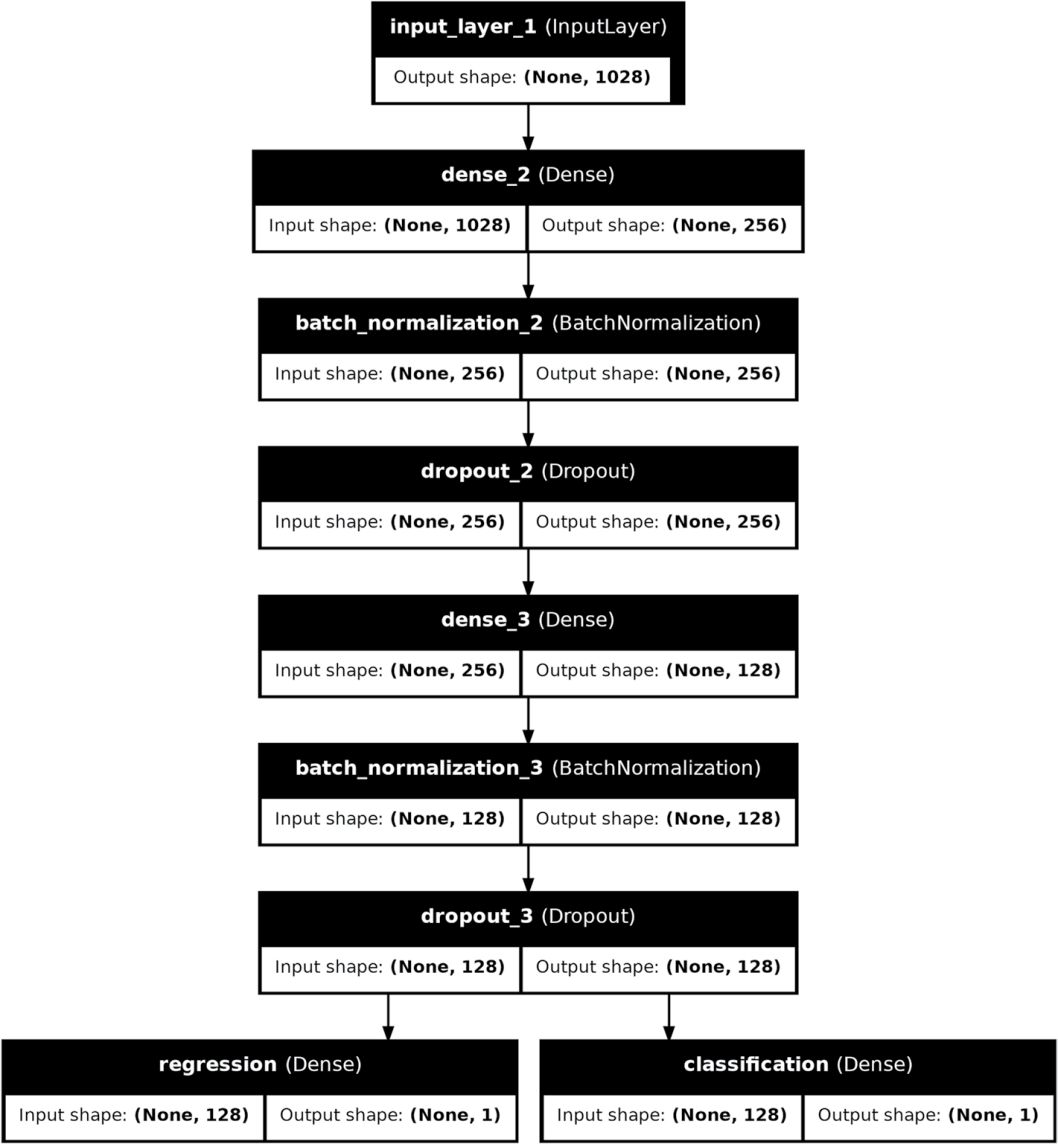
Neural network architecture

The model architecture consists of a shared base network composed of dense layers that capture general molecular patterns. This shared representation is then fed into two separate output heads–one dedicated to regression tasks (predicting pIC$_{50}$) and the other for classification tasks (predicting bioactivity classes). The architecture is shown in Fig. 3.

The model was optimized using task-specific loss functions, with mean squared error loss applied to the regression head and binary cross-entropy loss employed for the classification head.

During the model training phase, the dataset is split into training and validation sets to assess generalization. We implement early stopping and learning rate reduction callbacks to prevent overfitting, ensuring optimal training convergence.

### Performance metrics

We investigate various accuracy metrics used to evaluate the proposed models. Regarding the Regression approach, model performance is assessed using Mean Squared Error (MSE) and the $\hbox {R}^{2}$ score. Cross-validation is performed to ensure the generalizability of the models. We summarize the formal definitions of the aforementioned metrics in Table 3. We conduct a SHAP (SHapley Additive exPlanations) analysis to enhance interpretability. SHAP values are computed to quantify the impact of individual features on model predictions. Summary plots are generated to visualize feature importance, providing insights into how molecular descriptors influence the predicted pIC$_{50}$ values.

**Table 3:** Selected regression evaluation metrics, their formulas, and symbols

Performance Metric	Equation
Root Mean Squared Error (RMSE)	$\text {RMSE}=\sqrt{\frac{1}{T} \sum _{t=1}^{T} ( i_{t}-\hat{i}_{t})^2}$
Mean Squared Error (MSE)	$\text {MSE}=\frac{1}{T} \sum _{t=1}^{T} ( i_{t}-\hat{i}_{t})^2$
Coefficient of determination ($R^2$) [40]	$R^2= 1- \frac{\sum _{t=1}^{T} ( i_{t}- \hat{i}_{t})^2}{\sum _{t=1}^{T} ( i_{t}- \bar{i})^2}$

Symbols: *T*: Number of compounds, $i_{t}$: True value for compound t, $\hat{i}_{t}$: Predicted value for compound t, $\bar{i}:$ mean value of i

**Table 4:** Selected classification evaluation metrics and their formulas

Performance metric	Equation
Precision	$\text {Precision}= \frac{\text {True Positives}}{\text {True Positives} + \text {False Positives}}$
Recall	$\text {Recall}=\frac{\text {True Positives}}{\text {True Positives} + \text {False Negatives}}$
Accuracy	$\text {Accuracy} = \frac{\text {True Positives} + \text {True Negatives}}{\text {True Positives} + \text {True Negatives} + \text {False Positives} + \text {False Negatives}}$
$F_1$ score ($F_1$)	$F_1=2 \frac{\text {Precision} \times \text {Recall}}{\text {Precision} + \text {Recall}}$
AUC (Area Under the Curve)	$\text {AUC} = \int _{0}^{1} \text {TPR}(x) \,dx$

In the classification approach, we consider accuracy, precision, recall, F1 score, and Area Under the Curve (AUC) as classification accuracy metrics. We summarize the formal definitions of these metrics in Table 4.

In addition, we use the confusion matrix as a visual evaluation tool to reflect the classifier’s recognition ability for each class (Active, Inactive). We also use ROC (Receiver Operating Characteristic) curves and precision-recall curves to analyze each model’s predictive power. Furthermore, we compute cross-validation scores to ensure the robustness and generalizability of the trained classifiers.

### Bioactivity prediction web application

To facilitate the rapid assessment of molecular bioactivity, we develop a web-based application using Streamlit, integrating ML models with cheminformatics tools. The application predicts the bioactivity of compounds based on their Simplified Molecular Input Line Entry System (SMILES) representation. It provides predictions using a MTNN for $IC_{50}$ estimation and a Decision Tree classifier for categorical bioactivity classification.

The web application is accessible at: https://covid-19-mtnn.streamlit.app/.

#### Application workflow

**Fig. 4:**
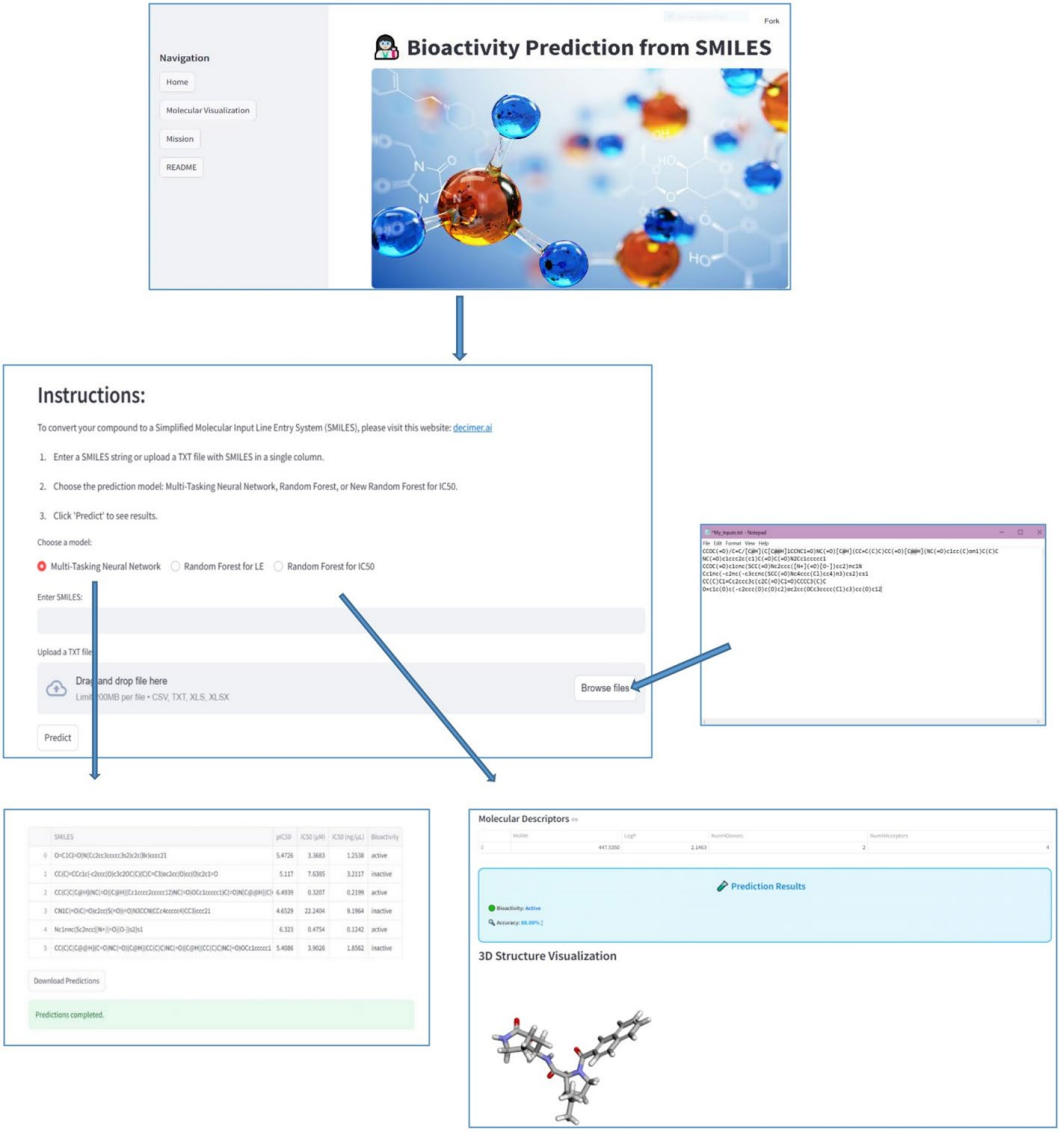
Workflow of the bioactivity prediction application

The application follows a structured workflow, as illustrated in Fig. 4:

1. *User Input* The user inputs a SMILES string manually or uploads a file containing multiple SMILES representations.
2. *Feature Extraction* The system computes molecular descriptors (e.g., molecular weight, LogP, hydrogen bond donors, hydrogen bond acceptors) and generates Morgan fingerprints using RDKit.
3. *Model Prediction* The user selects one of the two available predictive models:
  The Multi-task neural network (MTNN) estimates $IC_{50}$ values and predicts bioactivity.
  The Decision Tree classifier categorizes compounds into bioactivity classes (active, inactive).
4. *Results Display* The predicted values are displayed in a structured format, with an option to download the results as a CSV file for further analysis. Additionally, we have integrated a 3D visualization feature for SMILES representations, enabling users to explore molecular structures interactively, enhancing interpretability, and facilitating in-depth analysis.

#### Step-by-step usage instructions

The following steps should be followed to use the web application for predicting $IC_{50}$ values or classifying bioactivity:

1. Navigate to the web application interface at https://covid-19-mtnn.streamlit.app/.
2. Enter a SMILES string or upload a text file containing multiple SMILES.
3. Select the prediction model (MTNN for $IC_{50}$ prediction or Decision Tree for bioactivity classification).
4. Click the “Predict” button to initiate the analysis.
5. View the results on the interface, including molecular descriptors and predicted $IC_{50}$ values or bioactivity classes.
6. Download the prediction results for further research and analysis.

The web interface is designed for ease of use, featuring an intuitive layout with interactive components. The sidebar provides detailed descriptions of the predictive models, and results are dynamically updated based on user input. The application also supports batch processing of multiple compounds, making it a valuable tool for cheminformatics research and early-stage drug discovery.

## Results and discussions

This section presents the regression and classification accuracy results of the proposed approaches. Subsections “Regression results” and “Classification results” show the experimental results of the proposed regression and classification approaches. Also, Section “Multi-task neural network results” illustrates the experimental results of using the Multi-task Neural Network approach.

### Regression results

In this section, we evaluate the performance of three ML regression models: (i) Random Forest, (ii) Gradient Boosting, and (iii) XGB. Each algorithm is assessed individually, as well as in an ensemble that combines the three models. The evaluation is conducted using a dataset of molecular features, both with and without feature selection (FS) using LassoCV. All models, including the ensemble, are optimized through hyperparameter tuning. The best hyperparameters for the top-performing models are determined via extensive grid search and cross-validation. The final results are presented in Table 5, and the optimal parameters are summarized in Table 6.

**Table 5:** Comparison of Model Performance with and without Feature Selection Using Boosting Technique for Ensample Learning

Algorithm	Without FS			With FS		
	$\hbox {R}^{2}$	MSE	Training Time (s)	$\hbox {R}^{2}$	MSE	Training Time (s)
Random Forest	0.70	0.49	35.13	0.71	0.38	12.02
Gradient Boosting	0.70	0.49	7.35	0.73	0.36	2.47
XGBoost	0.71	0.48	17.25	0.72	0.37	5.92
Ensemble Model	0.71	0.38	29	0.73	0.36	9.91

**Table 6:** Hyperparameters of Machine Learning Models

Model	Hyperparameters	Grid search time (Sec.)
Random Forest	{max_depth: None, min_samples_leaf: 1, min_samples_split: 2, n_estimators: 400}	852.53
Gradient Boosting	{learning_rate: 0.1, max_depth: 4, n_estimators: 300}	211.00
XGBoost	{learning_rate: 0.1, max_depth: 4, n_estimators: 300}	184.57

As shown by the experimental results, XGB achieves the highest predictive accuracy among the individual models, yielding the lowest MSE (0.48) and the highest $\hbox {R}^{2}$ score (0.71). This suggests that XGB captures complex feature interactions slightly more effectively than Random Forest and Gradient Boosting. Feature selection (FS) using LassoCV enhances the performance of all three algorithms by eliminating irrelevant features.

In addition to performance improvements, FS significantly reduces the training time for most models, as shown in Table 5. The Random Forest’s training time drops from 35.13 to 12.02 s, and the Gradient Boosting model’s time decreases from 7.35 to 2.47 s. Similarly, XGB’s training time reduces from 17.25 to 5.92 s, likely due to its efficiency in handling a reduced feature space. These results highlight the computational advantages of FS, particularly for models that are sensitive to feature dimensionality.

Combining all three algorithms, the ensemble model without FS outperforms the individual models, demonstrating that integrating multiple learners enhances predictive power and reduces variance. As shown in Table 5, the ensemble model’s MSE improves from 0.38 to 0.36 after applying FS, while the $\hbox {R}^{2}$ score increases from 0.71 to 0.73. These results indicate that FS contributes to more accurate and efficient models.

Nevertheless, when feature selection (FS) is applied, Gradient Boosting performs comparably to the ensemble model while requiring substantially less training time (2.47 s vs. 9.91 s). This reduced computational cost, combined with similar predictive accuracy, makes Gradient Boosting with FS a less complex and more efficient choice. Therefore, considering the trade-off between accuracy and computational efficiency, Gradient Boosting with feature selection is identified as the preferred regression model in this study.

In addition, we evaluate various regression models to predict pIC$_{50}$ values based on molecular descriptors and fingerprints derived from SMILES data, as shown in Table 7. The performance of each model is assessed using MSE, RMSE, and R$^{2}$ score. The ensemble model combines the predictions of three algorithms: Random Forest, Gradient Boosting, and XGBoost.

**Table 7:** Comparison of different machine learning models based on MSE, RMSE, and$\hbox {R}^{2}$Score using FS

Model	MSE	RMSE	$R^2$Score
Linear Regression	0.40	0.63	0.70
Ridge Regression	0.40	0.63	0.70
Lasso Regression	1.33	1.15	− 0.00
ElasticNet Regression	1.30	1.14	0.02
Random Forest	0.38	0.62	0.71
AdaBoost	0.93	0.96	0.30
Support Vector Regressor	0.38	0.62	0.71
XGBoost	0.37	0.61	0.72
K-Neighbors Regressor	0.41	0.64	0.69
Gradient Boosting	0.36	0.62	0.73
Ensemble Model	0.36	0.62	0.73

**Fig. 5:**
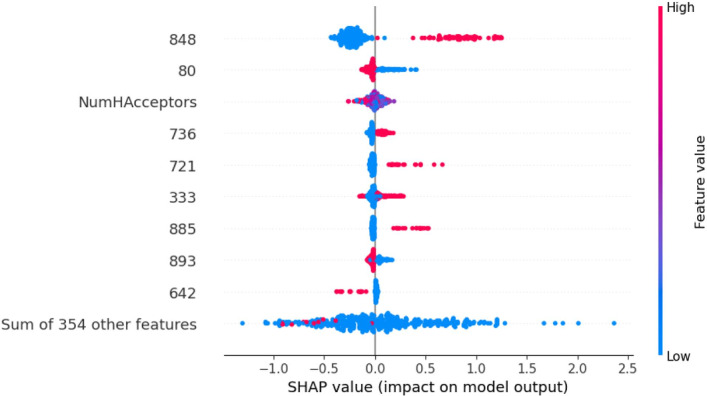
SHAP summary plot showing the contribution of each feature to the ensemble model’s predictions

To better understand the contribution of individual features to the model’s predictions, we generate a SHAP summary plot for the ensemble model with feature selection (FS), as shown in Fig. 5. This plot highlights the most influential molecular descriptors and fingerprints affecting pIC$_{50}$ predictions.

Features with higher SHAP values indicate a stronger impact on the model’s output. The plot reveals that the top-ranked features play the most significant roles in prediction, suggesting their potential biological relevance in determining compound activity.

Additionally, the plot includes a category labeled “sum of 345 other features”. This grouping represents features that, while still contributing to the model’s predictions, exhibit individually lower SHAP values. For clarity and visualization purposes, these features are aggregated rather than shown separately. Although each contributes a small effect, their combined influence may still have a meaningful impact on the overall prediction.

The distribution of SHAP values further reveals whether each feature increases or decreases the predicted pIC$_{50}$ values, thereby enhancing the interpretability of the model’s decision-making process.

### Classification results

This section presents the results of classification models used to predict the bioactivity status (active/inactive) of compounds based on molecular descriptors and fingerprints derived from SMILES data. Classification performance is evaluated using standard metrics, including accuracy, precision, recall, F1 score, and AUC. The results are organized into two subsections: IC$_{50}$-based bioactivity classification and LE-based bioactivity classification.

#### IC$_{50}$-based bioactivity classification results

IC50 is a widely used metric in bioactivity prediction, representing the compound concentration required to inhibit a biological process by 50%. This subsection analyzes the classification models applied to IC50-based bioactivity prediction.

Table 8 presents the classification performance of different models. The Random Forest classifier achieves the highest accuracy (88.09%) and AUC (0.9334), suggesting strong generalization performance. Gradient Boosting also performs well with an AUC of 0.9364, indicating high predictive capability. Logistic Regression, AdaBoost, and the MLP classifier demonstrate comparable performance, with an accuracy of around 86.15%.

**Table 8:** Comparison of different machine learning models based on Accuracy, Precision, Recall, and AUC on IC$_{50}$-Based Classification. The best-performing model is highlighted

Model	Accuracy	Precision	Recall	F1-score	AUC
Logistic Regression	0.8615	0.8613	0.8615	0.8610	0.9278
Support Vector Machine	0.5596	0.4773	0.5596	0.4379	0.5837
Decision Tree	0.8421	0.8417	0.8421	0.8417	0.8457
Random Forest	0.8809	0.8813	0.8809	0.8802	0.9334
Gradient Boosting	0.8615	0.8621	0.8615	0.8605	0.9364
AdaBoost	0.8504	0.8502	0.8504	0.8503	0.9097
K-Neighbors	0.7479	0.7519	0.7479	0.7489	0.8450
Naive Bayes	0.7701	0.7766	0.7701	0.7712	0.7805
MLP Classifier	0.8615	0.8613	0.8615	0.8610	0.9198

In contrast, the Support Vector Machine (SVM) performs poorly, with an accuracy of only 55.96%, likely due to the high dimensionality of the feature space and the presence of non-linear decision boundaries. Naive Bayes and K-Neighbors also exhibit relatively lower accuracy, suggesting their limited effectiveness for this classification task.

IC$_{50}$-based classification is a widely used approach for drug bioactivity prediction. ML models can leverage this classification to predict the activity of novel compounds, aiding in the early stages of drug discovery. Our results indicate that tree-based ensemble models, such as Random Forest and Gradient Boosting, perform best for this task. In the following subsection, we explore the integration of alternative bioactivity criteria, such as LE, to improve model robustness and drug-likeness predictions.

#### LE-based bioactivity classification results

LE is another key bioactivity criterion in drug discovery. It measures the binding efficiency of a compound relative to its molecular size. The classification models evaluated in this subsection focus on predicting bioactivity based on LE values.

Table 9 summarizes the classification performance of the models for LE-based prediction. Similar to IC$_{50}$ classification, models such as Random Forest, Gradient Boosting, and AdaBoost demonstrate strong predictive capabilities, outperforming simpler models. The Random Forest model again achieves the highest accuracy (0.917), reinforcing its effectiveness in bioactivity classification tasks.

**Table 9:** Comparison of different machine learning models based on Accuracy, Precision, Recall, and AUC on LE-Based Classification. The best-performing model is highlighted

Model	Training time (s)	Accuracy	Precision	Recall	F1 score	AUC
Logistic Regression	3.8903	0.8868	0.8866	0.8868	0.8865	0.9666
Support Vector Machine	6.1579	0.5811	0.6167	0.5811	0.4376	0.7635
Decision Tree	0.2846	0.8491	0.8495	0.8491	0.8492	0.8547
Random Forest	0.8938	0.9170	0.9173	0.9170	0.9171	0.9620
Gradient Boosting	3.3677	0.8792	0.8792	0.8792	0.8787	0.9587
AdaBoost	2.2431	0.8566	0.8566	0.8566	0.8558	0.9132
K-Neighbors	0.0146	0.7962	0.7954	0.7962	0.7954	0.8513
Naive Bayes	0.0492	0.8038	0.8043	0.8038	0.8040	0.8336
MLP Classifier	16.5930	0.8830	0.8830	0.8830	0.8829	0.9470

Logistic Regression, Gradient Boosting, and AdaBoost also perform well, with accuracy values of 0.886792, 0.8792, and 0.8566, respectively. These results indicate that ML-based models provide better classification accuracy than simpler models such as Decision Tree or Naive Bayes.

These findings highlight the significance of the Random Forest model in bioactivity classification and provide insights into the performance variations between IC$_{50}$-based and LE-based predictions.

**Fig. 6:**
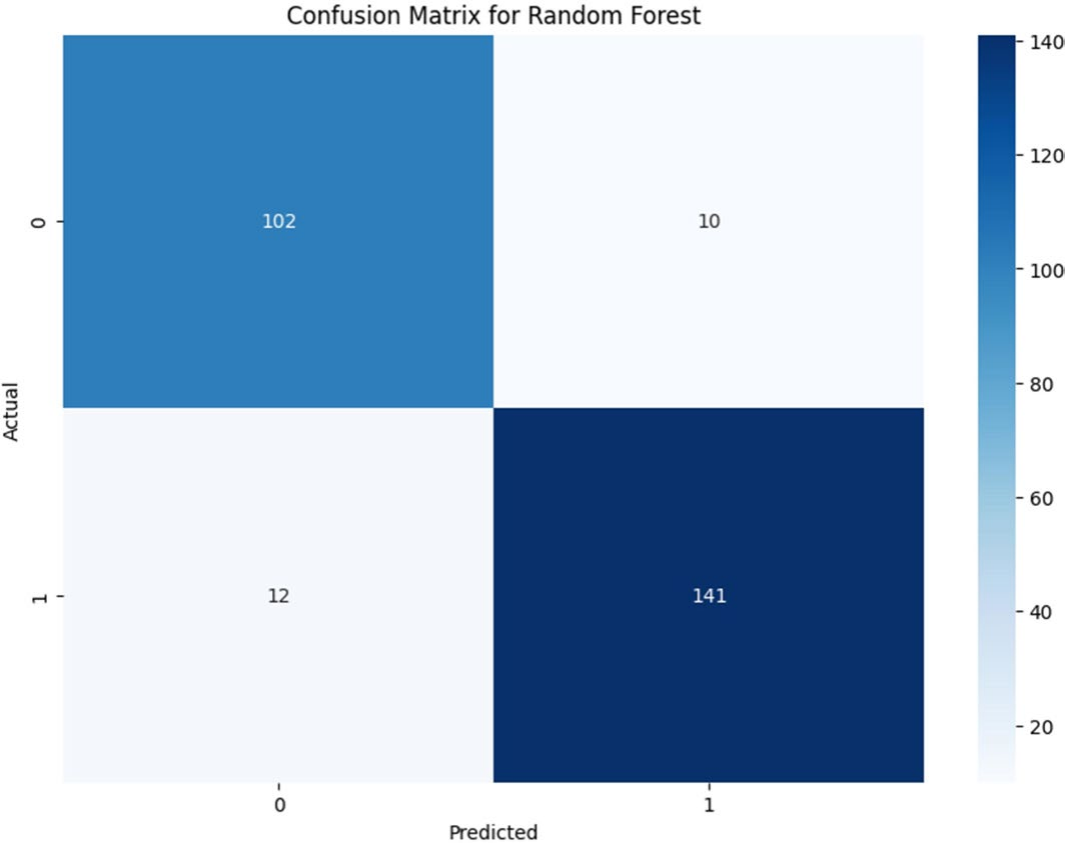
Confusion Matrix for LE-based Random Forest classification model

To further evaluate the classifiers, we use the confusion matrix. Figure 6 presents the confusion matrix for the Random Forest model. The diagonal elements represent correctly classified instances, while the off-diagonal elements indicate misclassifications. With an accuracy of 0.916981, the Random Forest model demonstrates strong classification performance, though some misclassifications exist.

**Fig. 7:**
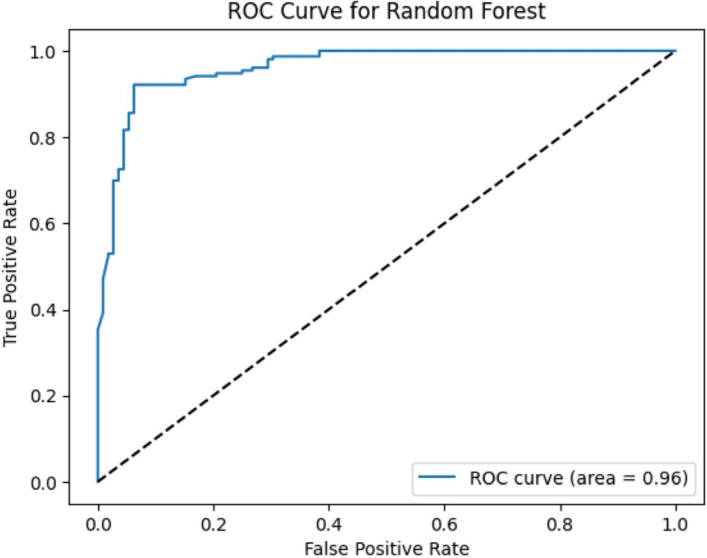
ROC Curve for LE-Based Random Forest classification model

Figure 7 shows the ROC curve for the Random Forest model. The curve demonstrates the model’s ability to distinguish between active and inactive compounds. With an AUC score of 0.962010, the model shows excellent class separation capabilities.

**Fig. 8:**
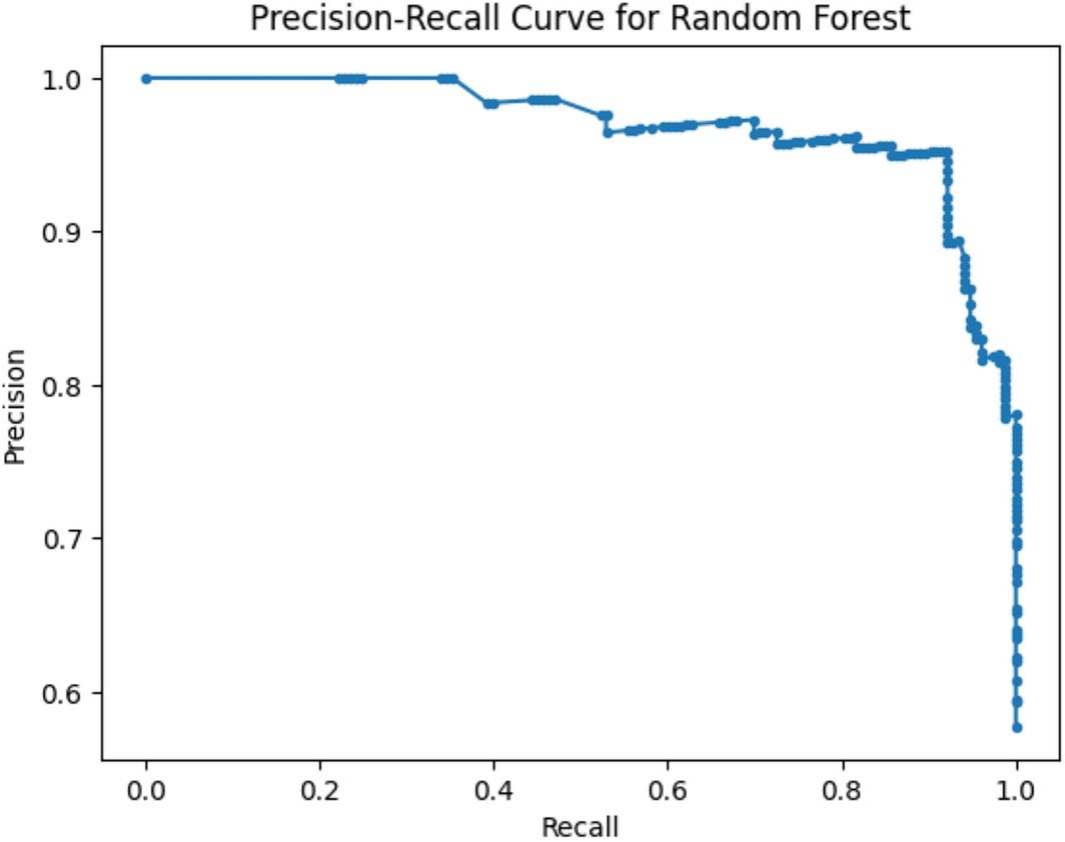
Precision-Recall Curve for LE-Based Random Forest classification model

Additionally, Fig. 8 presents the precision-recall curve for the Random Forest model. The curve remains close to the upper-right corner, indicating high precision and recall values, which confirm the model’s ability to correctly classify active compounds while minimizing false positives.

In summary, the Random Forest model emerges as the best-performing classifier with an accuracy of 0.917 and an AUC of 0.962, followed by Logistic Regression, Gradient Boosting, and AdaBoost, which also demonstrate strong predictive power. Although the Decision Tree model performs well, it does not achieve perfect classification performance as previously assumed. Methods such as Random Forest and Gradient Boosting significantly improve classification results, making them preferable choices for predicting compound activity status.

### Applicability domain analysis using Tanimoto similarity

To evaluate whether the predicted compounds were represented within the chemical space covered by the training set, an applicability domain (AD) analysis was performed using Tanimoto similarity calculated from Morgan fingerprints (radius = 2) [41, 42]. Following previous studies, a Tanimoto similarity threshold of 0.40 was used to define the AD boundary, whereby compounds with a maximum similarity score *\ge* 0.40 to at least one training compound were considered within the AD.

**Fig. 9:**
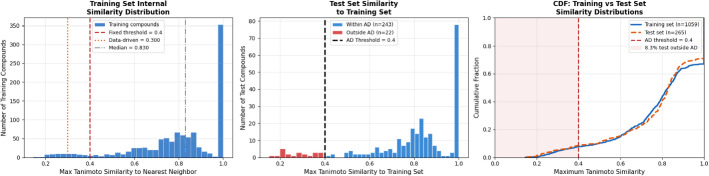
Distribution of maximum Tanimoto similarity values between test compounds and the training set. The dashed vertical line indicates the applicability domain threshold of 0.40. Most test compounds exhibit similarity values above the threshold, demonstrating substantial overlap between the training and test chemical spaces and supporting the reliability of the model predictions

The analysis showed that 243 of 265 compounds (91.7%) were located within the applicability domain (Fig. 9), indicating that the vast majority of predicted compounds share structural similarity with molecules present in the training set. This result suggests that most predictions were generated within the model’s learned chemical space rather than through extrapolation to structurally unrelated compounds.

The high AD coverage (91.7%) indicates that the model was applied predominantly to compounds structurally related to those used during training, supporting the reliability and interpretability of the generated predictions.

### Multi-task neural network results

This section presents the results of the MTNN on the validation set, as summarized in Table 10. The results compare the model’s performance with and without FS across regression and classification tasks. In general, adding FS leads to a notable improvement in model performance.

For the regression task, the model achieves an $\hbox {R}^{2}$ value of 0.77 with FS, compared to 0.69 without FS, indicating a stronger fit to the data. The regression loss, MSE, and RMSE also improve significantly, suggesting that the model’s predictions are more accurate when FS is applied. The decrease in MAE from 0.45 to 0.40 further highlights the benefit of FS in reducing prediction errors. These improvements suggest that FS helps eliminate irrelevant or redundant features, allowing the model to focus on more informative attributes.

**Table 10:** Validation Results of MTNN with and without FS

Metric	Without FS	With FS
Regression Loss	0.414438	0.303728
Regression MAE	0.448756	0.401997
Regression $\hbox {R}^{2}$	0.685745	0.771668
Regression MSE	0.417539	0.303376
Regression RMSE	0.646172	0.550796
Classification Loss	0.529138	0.418983
Classification Accuracy	0.872576	0.889197
Classification F1 score	0.870056	0.886364
Classification Precision	0.885057	0.906977
Classification Recall	0.855556	0.866667
Classification AUC	0.925246	0.940378
Training Time (Sec.)	234.3	205.35
Epochs	495	527

In the classification task, the model’s performance also improves with FS. The classification loss decreases from 0.53 to 0.42, and the accuracy increases from 87.26 to 88.92%. The F1 score, precision, and recall also improve, with the F1 score reaching 0.89. The AUC score increases from 0.93 to 0.94, indicating an enhanced ability to distinguish between active and inactive compounds. The increase in precision (from 0.89 to 0.91) suggests that the model makes fewer false positive predictions, while the slight improvement in recall (from 0.86 to 0.87) indicates that it is also better at detecting active compounds.

Additionally, FS results in a reduction in training time from 234.3 to 205.35 s. Despite requiring slightly more epochs (527 vs. 495), the overall training efficiency improves, suggesting that FS helps the model converge faster and generalize better.

**Fig. 10:**
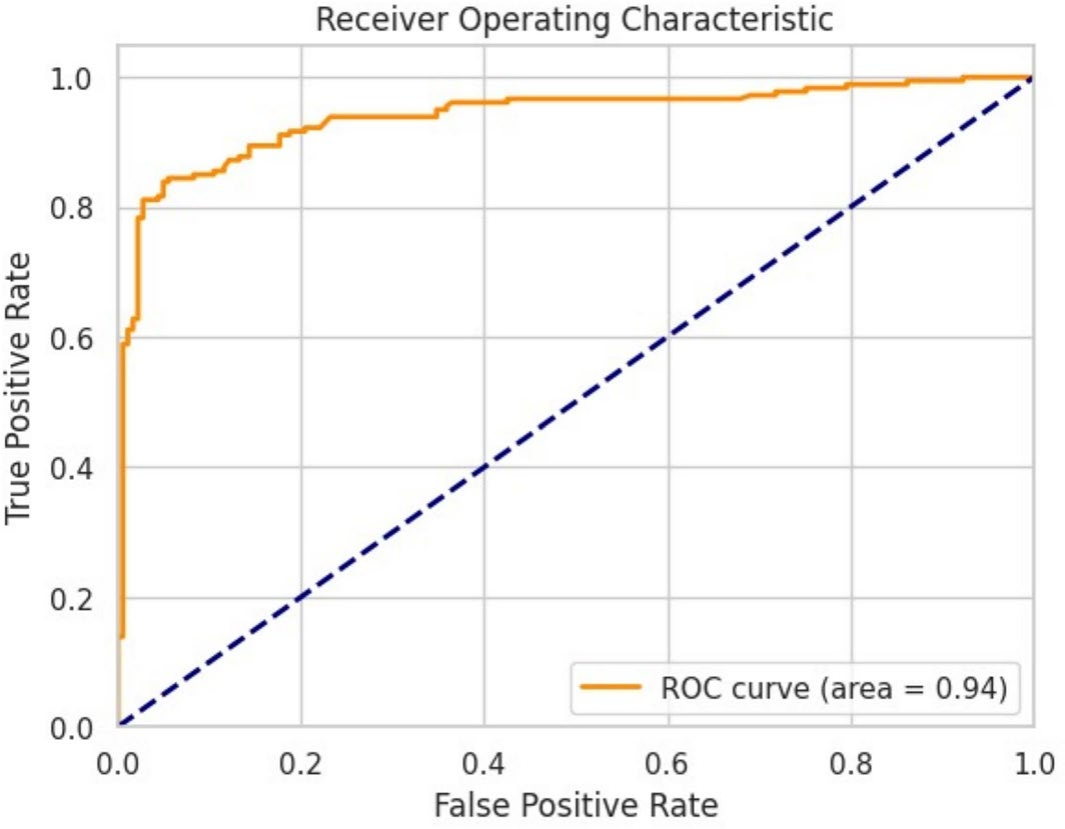
ROC Curve for Classification Task

Figure 10 shows the ROC curve for the classification task, with an AUC of 0.94, indicating strong discriminative ability. The model’s ROC curve (in dark blue) demonstrates a clear trade-off between the True Positive Rate (TPR) and the False Positive Rate (FPR), reflecting its ability to correctly classify positive instances while maintaining a low rate of false positives. The dashed line represents the random guess baseline (AUC = 0.5), and the model’s ROC curve is significantly above this, confirming its effectiveness. The improvement in AUC from 0.925 (without FS) to 0.94 (with FS) highlights the advantage of feature selection in enhancing classification performance.

**Fig. 11:**
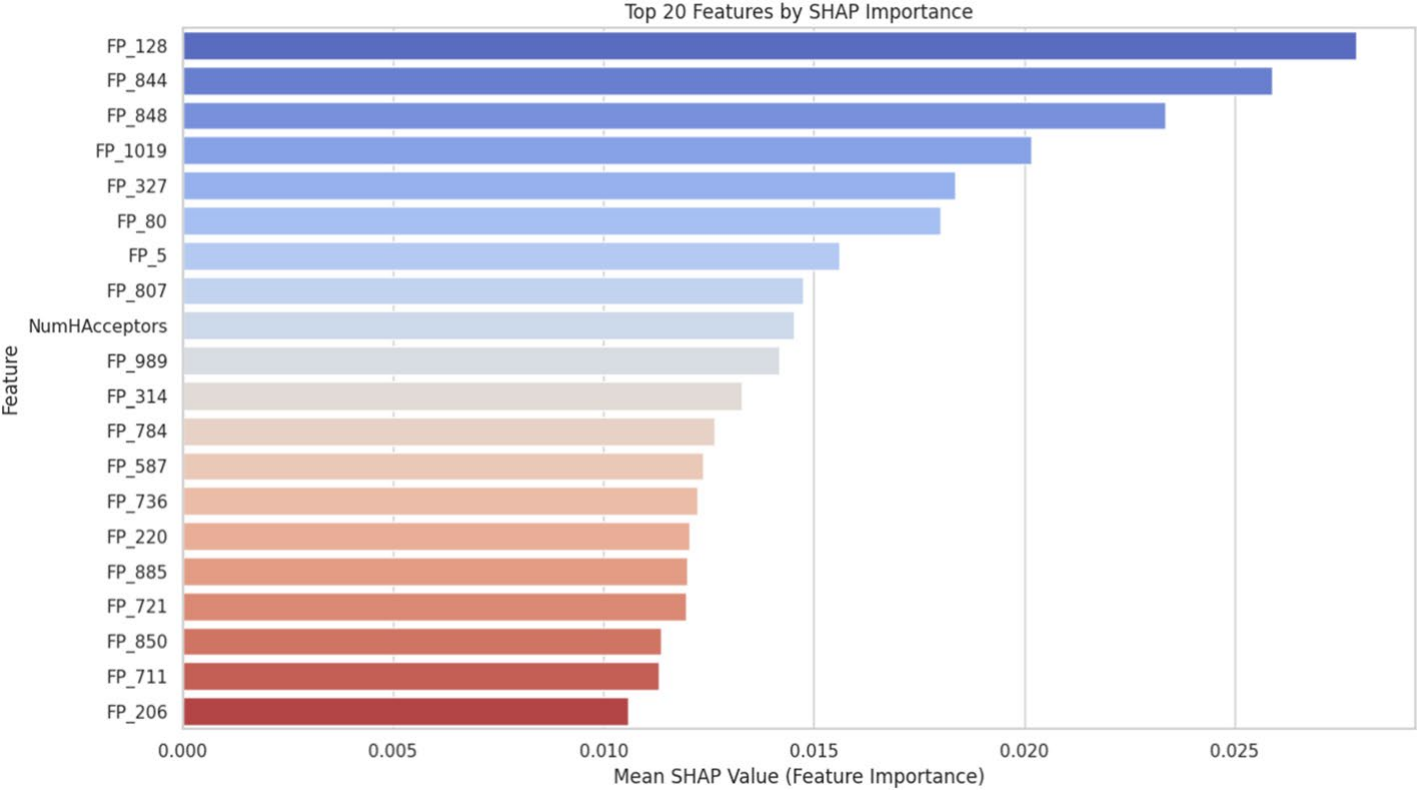
Top 20 Features by SHAP Importance

SHAP values are used to interpret the feature importance for the classification task. SHAP values provide a consistent and theoretically sound method to explain the output of the model by attributing the contribution of each feature. The mean absolute SHAP values are computed to rank the importance of features, and the top 20 features are shown in Fig. 11 and Table 11. The results indicate that certain features, including several Morgan fingerprints, are highly influential in the model’s predictions.

**Table 11:** Top 20 important features based on SHAP

Feature	SHAP Importance
FP_128	0.027907
FP_844	0.025896
FP_848	0.023363
FP_1019	0.020180
FP_327	0.018371
FP_80	0.018002
FP_5	0.015600
FP_807	0.014741
NumHAcceptors	0.014526
FP_989	0.014177
FP_314	0.013290
FP_784	0.012629
FP_587	0.012357
FP_736	0.012222
FP_220	0.012038
FP_885	0.011995
FP_721	0.011965
FP_850	0.011368
FP_711	0.011324
FP_206	0.010581

**Fig. 12:**
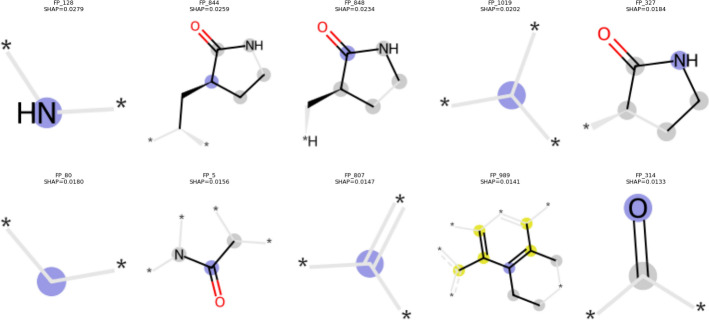
Structural back-projection of the top SHAP-ranked Morgan fingerprint bits. Representative molecular environments corresponding to the most influential fingerprint features identified by SHAP analysis are highlighted using RDKit. The associated SHAP values indicate the relative contribution of each fingerprint feature to model predictions

To enhance the interpretability of the SHAP analysis, the most influential Morgan fingerprint bits were mapped back to their corresponding molecular environments using RDKit fingerprint bit visualization. Representative molecular substructures associated with the top-ranked fingerprint bits are shown in Fig. 12, enabling direct chemical interpretation of the fingerprint features contributing to model predictions. The identified molecular environments predominantly include nitrogen-containing heterocycles, carbonyl-containing fragments, and sulfur-containing functional groups, suggesting that these structural motifs contribute substantially to the model’s bioactivity predictions.

**Fig. 13:**
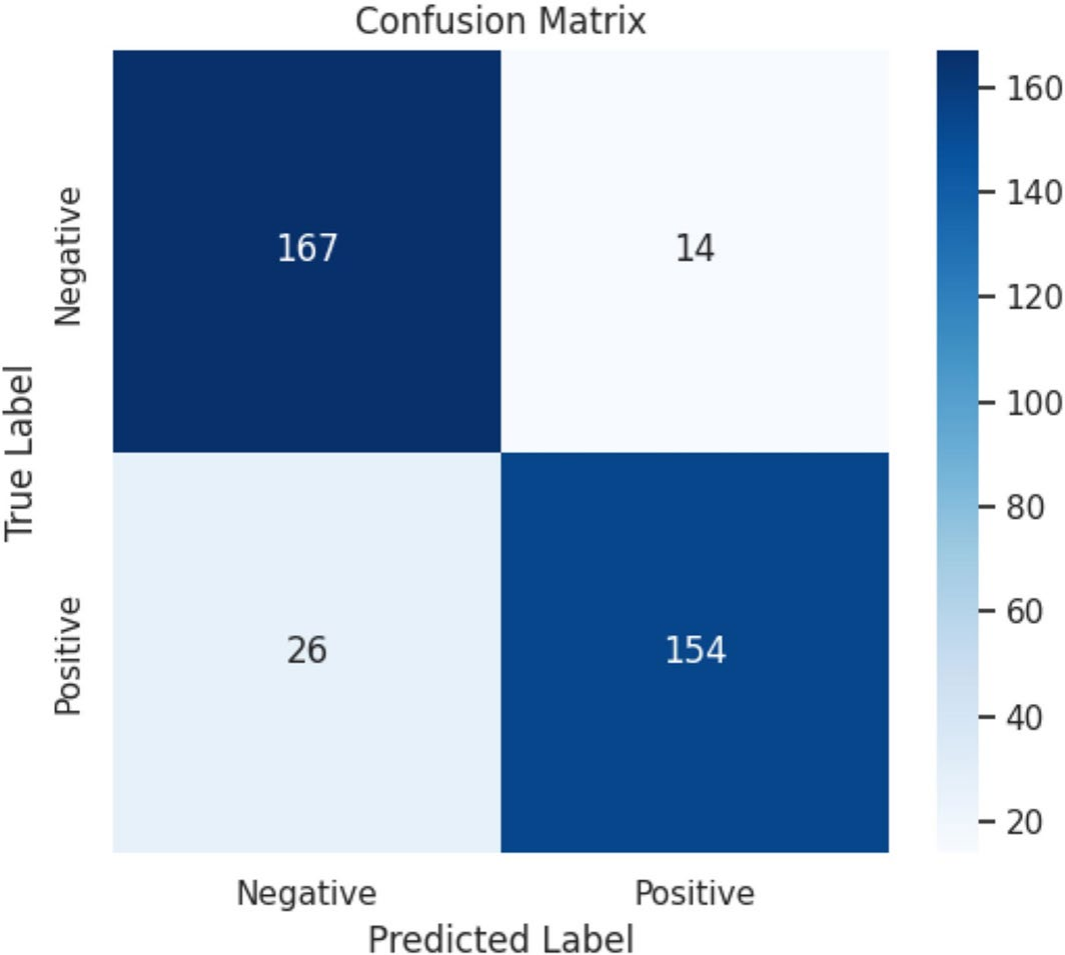
Confusion matrix for classification task

The confusion matrix, shown in Fig. 13, provides further insights into the model’s performance by illustrating the number of true positive, true negative, false positive, and false negative predictions. The confusion matrix helps in understanding the types of errors the model is making and in identifying areas for improvement.

Overall, FS enhances both regression and classification tasks, with notable gains in precision, F1 score, and AUC. This indicates a refined decision boundary that improves prediction accuracy. The reduction in training time further suggests that FS contributes to computational efficiency.

When compared to previous regression and classification approaches, the MTNN with FS demonstrates competitive performance. Gradient Boosting and the ensemble regression model with FS achieved an $\hbox {R}^{2}$ value of 0.73, which is slightly lower than the neural network’s $\hbox {R}^{2}$ value of 0.77, as shown in Table 5. For classification, traditional methods such as Random Forest and Gradient Boosting achieved classification accuracy values exceeding 0.91, outperforming the neural network, which achieved a classification accuracy of 91.7%. However, the neural network’s AUC of 0.94 remains strong, although slightly lower than the AUC of 0.96 achieved by the Random Forest model. This indicates that the Random Forest outperforms the neural network in terms of discriminating between the activity classes, as shown in Table 9.

### Validation of the proposed models

To validate the predictive capability of the proposed models, the predicted $IC_{50}$ values were compared against experimentally determined values reported by Jin et al. [43]. Table 12 summarizes this comparison, demonstrating the model’s performance across multiple reference compounds. For Ebselen, Tideglusib, and Carmofur, the predicted $IC_{50}$ values closely align with the corresponding experimental measurements, indicating that the MTNN effectively captures key molecular features influencing bioactivity. In contrast, larger deviations are observed for compounds such as Shikonin and PX-12, which may be attributed to higher experimental variability and increased molecular complexity.

Bioactivity classification was evaluated using three complementary approaches: MTNN-based classification, $IC_{50}$-based classification, and ligand efficiency (LE)-based classification. Ebselen is consistently classified as Active across all three models, reinforcing its strong inhibitory potential.

**Table 12:** Comparison of calculated for selected compounds from [43] and predicted $IC_{50}$ values using multi-task neural network model

Drug	Structure/SMILES	Calculated IC$_{50}$ (µM) [43]	Predicted IC$_{50}$ (µM)	MTNN-based Bioactivity Class	IC$_{50}$-based Bioactivity Class	LE-based Bioactivity Class
Ebselen	C1=CC=C(C=C1)N2C(=O)C3=CC=CC=C3[Se]2	0.67 ± 0.09	0.60	Active	Active	Active
Disulfiram	CCN(CC)C(=S)SSC(=S)N(CC)CC	9.35 ± 0.18	12.44	Inactive	Inactive	Active
Tideglusib	C1=CC=C(C=C1)CN2C(=O)N(C3=CC=CC4=CC=CC=C43)SC2=O	1.55 ± 0.30	0.43	Active	Inactive	Active
Carmofur	CCCCCCNC(=O)N1C=C(C(=O)NC1=O)F	1.82 ± 0.06	1.21	Active	Inactive	Active
Shikonin	CC(=CC[C@H](C1=CC(=O)C2=C(C=CC(=C2C1=O)O)O)O)C	15.75 ± 8.22	2.84	Inactive	Inactive	Inactive
PX-12	CCC(C)SSC1=NC=CN1	21.39 ± 7.06	3.25	Inactive	Inactive	Inactive

In contrast, Disulfiram, despite exhibiting a moderate $IC_{50}$ value, is classified as Active only by the LE-based model, suggesting that ligand efficiency provides additional insight beyond absolute potency values. Similarly, Tideglusib and Carmofur are predicted as Active by the MTNN, whereas the $IC_{50}$-based classification assigns them to the Inactive class. These discrepancies highlight the limitations of relying on a single bioactivity criterion.

The observed variations in classification outcomes can be attributed to threshold sensitivity, ligand efficiency considerations, and differences in model learning behavior. While $IC_{50}$-based classification relies on fixed cutoffs, LE-based classification accounts for molecular size and binding efficiency, allowing certain compounds to be prioritized despite moderate $IC_{50}$ values. Moreover, the MTNN, trained on a diverse feature space, is capable of capturing activity patterns that extend beyond simple threshold-based rules. Together, these factors emphasize the importance of integrating multiple classification strategies to achieve a more comprehensive assessment of compound bioactivity.

Despite minor discrepancies, the strong agreement between predicted and experimental values for several key reference compounds supports the validity of the proposed model for $IC_{50}$ prediction. The integration of multiple bioactivity classification approaches further enhances robustness by providing complementary perspectives on compound effectiveness. Future work will focus on refining classification thresholds and incorporating additional biochemical interaction features to further improve predictive consistency.

A second validation study was performed using a set of 32 bicycloproline derivatives targeting the SARS-CoV-2 main protease (Mpro), originally reported by Costa et al. [44]. As shown in Table 13, the predicted $IC_{50}$ and pIC$_{50}$ values obtained using the MTNN exhibit strong agreement with experimental measurements. For example, compounds DS3, DS5, and DS21 display experimental $IC_{50}$ values of 0.0165, 0.0132, and 0.0076 µM, respectively, which are closely approximated by the corresponding predicted values.

Notably, all 32 bicycloproline derivatives are consistently classified as Active by the MTNN-based, $IC_{50}$-based Random Forest, and LE-based Random Forest classifiers. This uniform classification highlights strong agreement among the different modeling strategies and reinforces the potential of these compounds as viable SARS-CoV-2 Mpro inhibitors.

Overall, these validation results demonstrate that the proposed MTNN model generalizes well beyond the training data and maintains stable predictive performance across independent experimental datasets, supporting its applicability in virtual screening and lead optimization for antiviral drug discovery.

To further validate our model’s predictive capability, we assess a set of selected compounds reported by Liu et al. [45], as summarized in Table 14. The comparison of categorized bioactivity classes and predicted $IC_{50}$ values using our MTNN model confirms that all listed compounds were classified as Inactive across three classification models: MTNN-based, $IC_{50}$-based, and LE-based classification. The alignment between these classifications suggests the reliability of our approach in identifying inactive compounds, further strengthening the model’s discriminative power.

The results demonstrate that none of the compounds from Liu et al. exhibit significant inhibitory potential, as indicated by their consistently inactive classification. This suggests that the structural or physicochemical properties of these compounds do not favor strong target binding or inhibition, reinforcing the effectiveness of our classification thresholds. The agreement across different classification approaches underscores the robustness of our predictive framework, providing confidence in its application for screening and prioritization of drug-like candidates.

Overall, the results indicate that the MTNN model provides reliable estimates of $IC_{50}$ and pIC50 values while effectively categorizing bioactive compounds. These findings support the applicability of our computational approach in virtual screening and lead optimization, which are essential for accelerating drug discovery against emerging viral threats such as SARS-CoV-2.

## Conclusion and future work

In conclusion, this paper presents a comprehensive approach to enhancing the drug discovery process for COVID-19 by leveraging machine learning techniques to predict pIC50 values. Our study investigated regression, classification, and multi-task neural network models, all of which demonstrated promising results in accurately predicting compound potency and categorizing activity. These models offer a scalable, cost-effective alternative to traditional experimental methods, providing valuable insights for compound selection and prioritization in drug development. By integrating these techniques into a user-friendly web application, we empower researchers with an accessible tool to accelerate drug discovery efforts.

**Table 13:** Comparison of IC50 and pIC50 values for 32 bicycloproline derivatives targeting Mpro of SARS-CoV-2 from [44] with predicted values obtained using our multi-task neural network model

ID	SMILES	IC50 **(** ${\upmu }$ M) [44]	pIC50 [44]	Predicted $IC_{50}$ ** (** ${\upmu }$ M)	Predicted $pIC_{50}$	Predicted Bioactivity*
DS1	CC1(C)[C@H]2C(C(=O)N[C@H](C=O)C[C@@H]3CCNC3=O)N(C[C@H]21)C(=O)c1cc2ccccc2cc1	0.453	6.344	0.93	6.03	Active
DS2	CC1(C)[C@H]2C(C(=O)N[C@H](C=O)C[C@@H]3CCNC3=O)N(C[C@H]21)C(=O)COc1cc2ccccc2cc1	0.052	7.283	0.04	7.42	Active
DS3	CC1(C)[C@H]2C(C(=O)N[C@H](C=O)C[C@@H]3CCNC3=O)N(C[C@H]21)C(=O)CCc1cccc(F)c1	0.0165	7.783	0.02	7.69	Active
DS4	CC1(C)[C@@H]2C(C(=O)N[C@@H](C=O)C[C@@H]3CCNC3=O)N(C[C@@H]21)C(=O)COc1cc(F)cc(F)c1	0.185	7.733	0.03	7.50	Active
DS5	CC1(C)[C@H]2C(C(=O)N[C@H](C=O)C[C@@H]3CCNC3=O)N(C[C@H]21)C(=O)COc1ccc(F)c(F)c1	0.0132	7.879	0.02	7.76	Active
DS6	CC1(C)[C@@H]2C(C(=O)N[C@@H](C=O)C[C@H]3CCNC3=O)N(C[C@@H]21)C(=O)COc1ccc(OC)c(OC)c1	0.0145	7.839	0.02	7.62	Active
DS7	CC1(C)[C@H]2C(C(=O)N[C@H](C=O)C[C@@H]3CCNC3=O)N(C[C@H]21)C(=O)COc1cc(ccc1)C(F)(F)F	0.0433	7.364	0.03	7.57	Active
DS8	CC1(C)[C@@H]2C(C(=O)N[C@@H](C=O)C[C@H]3CCNC3=O)N(C[C@@H]21)C(=O)COc1ccc(OC)c(F)c1	0.0372	7.429	0.01	7.87	Active
DS9	CC1(C)[C@H]2C(C(=O)N[C@H](C=O)C[C@@H]3CCNC3=O)N(C[C@H]21)C(=O)COc1ccc(OC(F)(F)F)cc1	0.0152	7.818	0.02	7.77	Active
DS10	CC1(C)[C@@H]2C(C(=O)N[C@@H](C=O)C[C@H]3CCNC3=O)N(C[C@@H]21)C(=O)COc1ccc(Cl)c(Br)c1	0.0508	7.294	0.01	7.97	Active
DS11	CC1(C)[C@@H]2C(C(=O)N[C@@H](C=O)C[C@@H]3CCNC3=O)N(C[C@@H]21)C(=O)COc1ccc(Br)cc1Cl	0.0133	7.876	0.02	7.67	Active
DS12	CC1(C)[C@@H]2C(C(=O)N[C@@H](C=O)C[C@H]3CCNC3=O)N(C[C@@H]21)C(=O)COc1ccc(Cl)cc1	0.019	7.721	0.02	7.61	Active
DS13	CC1(C)[C@H]2C(C(=O)N[C@H](C=O)C[C@@H]3CCNC3=O)N(C[C@H]21)C(=O)COc1ccc(Cl)c(Cl)c1	0.0124	7.907	0.03	7.48	Active
DS14	CC1(C)[C@@H]2C(C(=O)N[C@@H](C=O)C[C@H]3CCNC3=O)N(C[C@@H]21)C(=O)COc1ccc(Cl)cc1Cl	0.013	7.886	0.03	7.48	Active
DS15	O=C1NCC[C@@H]1C[C@H](C=O)NC(=O)C1[C@H]2CCC[C@H]2CN1C(=O)c1cccnc1	0.7485	6.126	0.62	6.21	Active
DS16	O=C1NCC[C@@H]1C[C@H](C=O)NC(=O)C1[C@H]2CCC[C@H]2CN1C(=O)c1cc2ccccc2[NH]1	0.1531	6.815	0.09	7.07	Active
DS17	O=C1NCC[C@@H]1C[C@H](C=O)NC(=O)C1[C@H]2CCC[C@H]2CN1C(=O)c1cc2ccccn2n1	0.2988	6.525	0.24	6.61	Active
DS18	O=C1NCC[C@@H]1C[C@H](C=O)NC(=O)C1[C@H]2CCC[C@H]2CN1C(=O)/C=C/c1ccccc1	0.5259	6.279	0.27	6.57	Active
DS19	O=C1NCC[C@@H]1C[C@H](C=O)NC(=O)C1[C@H]2CCC[C@H]2CN1C(=O)/C=C/c1ccc(OC)cc1OC	0.1956	6.709	0.11	6.96	Active
DS20	CN(C)c1ccc(cc1)/C=C/C(=O)N1C[C@@H]2CCC[C@@H]2C1C(=O)N[C@@H](C=O)C[C@H]1CCNC1=O	0.375	6.426	0.32	6.49	Active
DS21	O=C1NCC[C@@H]1C[C@H](C=O)NC(=O)C1[C@H]2CCC[C@H]2CN1C(=O)CCc1cccc(F)c1	0.0076	8.119	0.02	7.73	Active
DS22	O=C1NCC[C@@H]1C[C@H](C=O)NC(=O)C1[C@H]2CCC[C@H]2CN1C(=O)CCc1ccc(F)c(F)c1	0.0174	7.759	0.02	7.61	Active
DS23	O=C1NCC[C@@H]1C[C@H](C=O)NC(=O)C1[C@H]2CCC[C@H]2CN1C(=O)CCc1cc(F)cc(F)c1	0.0076	8.119	0.06	7.21	Active
DS24	CN(C)c1ccc(cc1)CCC(=O)N1C[C@@H]2CCC[C@@H]2C1C(=O)N[C@@H](C=O)C[C@H]1CCNC1=O	0.3782	6.422	0.22	6.65	Active
DS25	O=C1NCC[C@@H]1C[C@H](C=O)NC(=O)C1[C@H]2CCC[C@H]2CN1C(=O)COc1ccc(OC)cc1	0.0362	7.441	0.03	7.48	Active
DS26	O=C1NCC[C@@H]1C[C@H](C=O)NC(=O)C1[C@H]2CCC[C@H]2CN1C(=O)C(C)Oc1ccc(OC)cc1	0.0691	7.161	0.11	6.94	Active
DS27	O=C1NCC[C@H]1C[C@@H](C=O)NC(=O)C1[C@@H]2CCC[C@@H]2CN1C(=O)COc1ccc2OCCOc2c1	0.0935	7.029	0.04	7.44	Active
DS28	O=C1NCC[C@H]1C[C@@H](C=O)NC(=O)C1[C@@H]2CCC[C@@H]2CN1C(=O)COc1ccc(Cl)cc1	0.0092	8.036	0.03	7.59	Active
DS29	O=C1NCC[C@H]1C[C@@H](C=O)NC(=O)C1[C@@H]2CCC[C@@H]2CN1C(=O)COc1ccc(Cl)cc1F	0.0347	7.460	0.02	7.78	Active
DS30	O=C1NCC[C@H]1C[C@@H](C=O)NC(=O)C1[C@@H]2CCC[C@@H]2CN1C(=O)COc1ccc(Cl)cc1Cl	0.0172	7.764	0.02	7.69	Active
DS31	O=C1NCC[C@H]1C[C@@H](C=O)NC(=O)C1[C@@H]2CCC[C@@H]2CN1C(=O)COc1ccc(Cl)c(Cl)c1	0.03	7.523	0.04	7.42	Active
DS32	O=C1NCC[C@H]1C[C@@H](C=O)NC(=O)C1[C@@H]2CCC[C@@H]2CN1C(=O)COc1ccc(Cl)cc1C	0.0197	7.706	0.02	7.67	Active

$^{*}$ The predicted bioactivity class is determined based on three models: (i) MTNN, (ii) $IC_{50}$-based Random Forest, and (iii) LE-based Random Forest, as shown in the last three columns of Table 12. All listed compounds are predicted to belong to the Active bioactivity class by all three classification models

**Table 14:** Comparison of categorized class for selected compounds from [45] and predicted $IC_{50}$ values using multi-task neural network model

Drug	Structure/SMILES	Bioactivity Class [45]	LE-based Class	$IC_{50}$-based Class
NT-1–21	CSC1=C(C=CC=N1)C#N	Inactive	Inactive	Inactive
NT-1–24	CS(=O)(=O)C1=C(C=CC=N1)C#N	Inactive	Inactive	Inactive
NT-1–32	CS(=O)(=O)C1=C(C=CC(=N1)C(F)(F)F)C#N	Inactive	Inactive	Inactive
AZVIII-44B	C1=CN=CC(=C1)CNS(=O)(=O)C2=CC=CN=C2	Inactive	Inactive	Inactive
AZVIII-44D	C1=CC=C(C=C1)CNS(=O)(=O)C2=CC=CC=C2	Inactive	Inactive	Inactive
AZVIII-57 G	COC1=CC=C(C=C1)S(=O)(=O)N(CC=O)CC2=CC=CC=C2	Inactive	inactive	inactive
AZVIII-49C	C1=CN=CC(=C1)S(=O)(=O)NCC2=NC=NC=C2	Inactive	Inactive	Inactive
AZVIII-44 H	COC1=CC=C(C=C1)CNS(=O)(=O)C2=CC=CN=C2	Inactive	Inactive	Inactive
AZVIII-44E	C1=CC=C(C=C1)CNS(=O)(=O)C2=CC=CN=C2	Inactive	Inactive	Inactive
AZVIII-57D	C1=CC=C(C=C1)CN(CC=O)S(=O)(=O)C2=CC=CC=C2	Inactive	Inactive	Inactive
AZVIII-49F	C1=CC=C(C=C1)S(=O)(=O)NCC2=NC=NC=C2	Inactive	Inactive	Inactive

The key results from our study reveal that the best performance for regression tasks was achieved using the multi-task neural network with feature selection (FS), which yielded an $\hbox {R}^{2}$ value of 0.77. This performance surpasses that of the individual Gradient Boosting algorithm combined with FS, which achieved an $\hbox {R}^{2}$ of 0.73, demonstrating the superior predictive power and robustness of the multi-task approach for pIC50 predictions. For the classification task, traditional methods such as Decision Trees, Gradient Boosting, and AdaBoost delivered near-perfect accuracy, precision, and recall (1.00), outperforming the neural network that achieved a classification accuracy of 88.92% but still maintained a strong AUC of 0.94.

Overall, integrating FS with these advanced machine learning models provides a powerful and efficient solution for predicting compound activity, significantly improving both accuracy and scalability compared to traditional methods. These findings underscore the importance of selecting and tailoring specific machine learning algorithms to the task at hand, thereby optimizing performance in drug discovery applications.

Future work should focus on exploring advanced deep learning architectures, such as graph neural networks and transformer-based models, and large language models (LLMs), to enhance molecular representations and capture intricate structural and semantic relationships. Integrating additional heterogeneous data sources, including high-throughput screening results and molecular dynamics simulations, can further enrich the feature space, while using data augmentation techniques like randomized SMILES may alleviate data scarcity and imbalance issues. Developing multi-objective optimization frameworks to concurrently predict compound potency, toxicity, and ADMET profiles will better reflect the multifaceted nature of drug discovery. Future work may also investigate the integration and comparison of additional molecular fingerprint representations, such as MACCS keys and RDKit fingerprints, to further enhance predictive performance and capture complementary structural features. Furthermore, incorporating explainable AI methods is essential to elucidate the model’s decision-making process and highlight critical molecular features. Finally, establishing iterative feedback loops that integrate computational predictions with experimental validations and fostering collaborations between academia and industry will be crucial to translating these computational advances into practical and scalable drug discovery workflows.

## Data Availability

The datasets analyzed in this study are publicly available from the ChEMBL database (https://www.ebi.ac.uk/chembl/). Data were collected for human-related coronaviruses and filtered to include targets classified as SINGLE PROTEIN or PROTEIN COMPLEX with experimentally determined IC50 values. The final dataset consists of 1,803 data points. The implementation code, trained models, and supporting resources used in this study are publicly available at: https://github.com/SohilaOsama/COVID-19
